# *ROI-Finder*: machine learning to guide region-of-interest scanning for X-ray fluorescence microscopy

**DOI:** 10.1107/S1600577522008876

**Published:** 2022-10-25

**Authors:** M. A. Z. Chowdhury, K. Ok, Y. Luo, Z. Liu, S. Chen, T. V. O’Halloran, R. Kettimuthu, A. Tekawade

**Affiliations:** a Data Science and Learning Division, Argonne National Laboratory, Lemont, IL 60439, USA; b Department of Microbiology and Molecular Genetics, Michigan State University, East Lansing, MI 48824, USA; c X-ray Science Division, Argonne National Laboratory, Lemont, IL 60439, USA; d Advanced Photon Source, Argonne National Laboratory, Lemont, IL 60439, USA; e Department of Chemistry, Michigan State University, East Lansing, MI 48824, USA; University of Essex, United Kingdom

**Keywords:** X-ray fluorescence, region-of-interest detection, principal components analysis, live cell imaging, microscopy, *E. coli*, fuzzy clustering

## Abstract

The microscopy research at the Bionanoprobe (currently at beamline 9-ID and later 2-ID after APS-U) of Argonne National Laboratory focuses on applying synchrotron X-ray fluorescence (XRF) techniques to obtain trace elemental mappings of cryogenic biological samples to gain insights about their role in critical biological activities. The elemental mappings and the morphological aspects of the biological samples, in this instance, the bacterium *Escherichia coli* (*E. Coli*), also serve as label-free biological fingerprints to identify *E. coli* cells that have been treated differently. The key limitations of achieving good identification performance are the extraction of cells from raw XRF measurements via binary conversion, definition of features, noise floor and proportion of cells treated differently in the measurement. Automating cell extraction from raw XRF measurements across different types of chemical treatment and the implementation of machine-learning models to distinguish cells from the background and their differing treatments are described. Principal components are calculated from domain knowledge specific features and clustered to distinguish healthy and poisoned cells from the background without manual annotation. The cells are ranked via fuzzy clustering to recommend regions of interest for automated experimentation. The effects of dwell time and the amount of data required on the usability of the software are also discussed.

## Abstract 2

The microscopy research at the Bionanoprobe (currently at beamline 9-ID and later 2-ID after APS-U) of Argonne National Laboratory focuses on applying synchrotron X-ray fluorescence techniques to obtain trace elemental mappings of cryogenic biological samples to gain insights about their role in critical biological activities.

## 1. Introduction

The Bionanoprobe (BNP) at beamline 9-ID of the Advanced Photon Source (APS) at Argonne National Laboratory focuses on advancing synchrotron-based X-ray fluorescence (XRF) imaging and elemental mapping techniques for a broad range of biological studies including the imaging of mouse fibroblast cells (Chen *et al.*, 2015 ▸), the effect of nanomedicine on rabbit liver tissues via sulfur maps (Deng *et al.*, 2022 ▸), a simultaneous qualitative investigation of structural features and quantitative elemental maps of *ex vivo* tissues (Genoud *et al.*, 2020 ▸), and specifying discrete zinc-enriched vesicles in oocyte growth and egg fertilization via single-cell level zinc mapping (Que *et al.*, 2015 ▸). Facilitation of further scientific discovery at the 9-ID beamline as well as other beamlines at the APS and their consequent scientific impact is expected to accelerate with the upcoming APS upgrade (APS-U) providing higher-energy X-rays for faster measurement and higher resolution (Fornek, 2019 ▸). The orders-of-magnitude increase in acquired XRF images necessitates integration of machine-learning methods for data analysis, smart experimentation, and autonomous decision-making for interpretation and exploration of high-dimensional parameter spaces.

The elemental maps that form the XRF images are typically scanned with coarse and fine resolution by raster-scanning a point probe at the BNP. From the coarse-resolution XRF images, several regions of interest (ROIs) are identified. The extracted ROIs could contain specific morphological and elemental information (*e.g.* the detection of individual biological cells) providing image-based features to fit clustering models. Use of multi-dimensional elemental maps to identify and locate ROIs from XRF images for cells of similar shape and morphology have been reported (Wang *et al.*, 2014 ▸). However, in practice, individual cells within a population can exhibit differing morphologies and elemental variations. The variations in morphology and elemental content can be utilized by machine-learning models to further isolate and precisely identify and locate cells. The prohibitively long acquisition time for fine-resolution scans and the lack of automation in locating ROIs in elemental maps produced in more rapid coarse scans constitute a major bottleneck in developing a quantitative understanding of the inorganic variations within a population of cells. Automation to cluster and detect ROIs has been attempted previously. A conventional hard *k*-means clustering algorithm alone may not be sufficient (Ward *et al.*, 2013 ▸). Mean shift clustering (Comaniciu & Meer, 2002 ▸) provides less control over the number of clusters and may provide abrupt changes to the number of clusters in high-dimensional data. Affinity propagation (Frey & Dueck, 2007 ▸) is quite memory intensive and thus does not scale well to large datasets. Density-based spectral clustering application with noise (DBSCAN; Ester *et al.*, 1996 ▸) is dependent on the ordering of data points and is highly sensitive to its hyperparameters, particularly the epsilon hyperparameter. While the challenge with ordering of points is addressed by the *OPTICS* (*ordering points to identify the clustering structure*) algorithm (Ankerst *et al.*, 1999 ▸), it still requires the number of minimum data points to be specified for clustering. The advantages of using *k*-means over the other algorithms are its simplicity and the choice of the number of clusters the user prefers in order to find the desired number of bacterial cell type treatments. To fully automate experimentation, soft or fuzzy clustering is required to rank and group cells (Li *et al.*, 2017 ▸). Furthermore, the user should also be provided with an interface to view clustered data based on the chosen features. Faster annotation could then be performed using, for example, a lasso-selector tool.

Open-source software provides the ability to rapidly switch between different algorithms and prototype new ones and is required for targeted experimentation. Therefore, stand-alone open source software packages for beamline scientists and users are needed to assist with fast installation, rapid learning curves, superior usability and integration of domain knowledge for specific machine-learning applications. In this paper, we report the development of the open-source software *ROI-Finder* and demonstrate its use for automating beam time experiments involving the imaging of *Escherichia coli* (*E. coli*) cells frozen on a grid after treatment with different poisoning reagents in order to understand if the inorganic content of individual cells can serve as a reporter of the physiological stress. XRF is a non-intrusive method for analyzing individual *E. coli* cells (Victor *et al.*, 2020 ▸). During the experiments reported here, a combination of coarse and fine measurements resulted in XRF maps of trace elements as well as morphological variations within numerous detected cells. During the beam time experiment, an unsupervised fuzzy clustering model was trained on the coarse scans to group and rank detected ROIs based on their similarity, and selected ROIs were recommended for subsequent high-resolution scanning. *ROI-Finder* was demonstrated to selectively image fine-resolution scans of healthy and poisoned *E. coli* cells imaged under cryogenic conditions in collaboration with the APS team who operated the instrument and the participating user who planned and prepared the samples for these experiments. Through further discussions, we show that the code can be used in multiple scenarios, including recommending similar cells, identifying live and dead cells, and similarity-based sorting by a fuzzy clustering algorithm to rapidly identify experimental anomalies.

**Figure 1:**
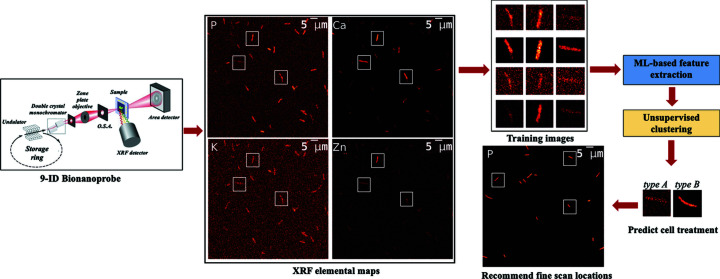
Schematic of the *ROI-Finder* workflow for similar cell recommendations and prediction of cell states. The acquired coarse scan microscopy elemental maps are segmented and individual cell images are extracted. *ROI-Finder* uses PCA to extract features from individual cell images. The features are fed to a fuzzy *k*-means unsupervised clustering algorithm to train the model. The trained model is used to predict cell treatment types and recommended scan locations for automated steering of experiments.

Fig. 1 ▸ provides an overview of the implementation of *ROI-Finder*. The XRF microscopy images acquired via coarse scans contain *E. coli* cells. From each elemental channel individual *E. coli* cells are extracted. From the cell images, cell morphology parameters are calculated and features for the principal component analysis (PCA) are extracted. The principal components (PCs) are used to train a fuzzy *k*-means clustering algorithm. Two possible scenarios for ROI identification are to distinguish between *E. coli* cells that have been treated differently or to recommend fine scan locations similar to those previously selected. From the fuzzy clustered space and domain heuristics, the model can predict type A (healthy) and type B (poisoned) *E. coli* cells to sort and rank them based on a confidence metric. The model recommends cells of similar morphology and elemental mappings from clustered feature space. The co-ordinates of the recommended ROIs are provided to the data acquisition and control system of the BNP to conduct fine-resolution scans on similar cells to gain detailed local elemental mappings on cells of interest.

## 2. Experimental

### 2.1. Sample preparation

Inorganic substances including transition metals play a pivotal role in living organisms and control challenging chemical processes required for the sustainability of life (O’Halloran, 1993 ▸; Pushie *et al.*, 2014 ▸). Microbes can respond to a limitation and excess of metal ions by activating the expression of metalloregulatory proteins and/or metal-specific receptors (Finney & O’Halloran, 2003 ▸; Chandrangsu *et al.*, 2017 ▸). Microbes also utilize inorganic substances as an essential regulator for life; therefore, identifying a biodistribution of inorganic trace elements in a single bacterial cell is of high interest (Williams, 2006 ▸). In these experiments, three different types of *E. coli* cells were prepared. A single colony of *E. coli* K-12 wild-type strain MG1655 was inoculated in 3 ml of sterilized LB broth (Miller’s modification, Sigma Aldrich) and incubated overnight (16 h) at 37°C with 250 rpm shaking. The overnight culture was diluted to 1:100 in the 5 ml of fresh LB media and grown to reach the early log phase (OD_6_00 = 0.3–0.4). *E. coli* cells were then exposed to treatment, incubated for another 20 min and transferred to metal-free conical tubes followed by three spin-down washes with 240 m*M* sucrose (Sigma Aldrich) solution. Three different treatments were performed: healthy untreated (type A), 70% ethanol poisoned (type B) and 1 m*M* sodium azide (NaN_3_, Sigma Aldrich) plus 0.1 m*M* copper sulfate (CuSO_4_, Sigma Aldrich) treated (type C). The culture prepared without any treatment are the type A cells. For type B cells, the LB medium was substituted with 5 ml of 70% (*v*/*v*) ethanol solution and incubated for 20 min to poison the early log phase culture. For type C cells, the treatment was performed using 1 m*M* NaN_3_ and 0.1 m*M* CuSO_4_ in early log phase culture. All washed samples were loaded on a 1.50 mm × 1.50 mm sized silicon nitride (SiN) window (Norcada, NX5150D) using a plunge freeze setup. Each sample (2 µl) was loaded in the middle of the SiN window and blotted by blot paper to evenly disperse on the surface. After, the SiN window was immediately dipped into liquefied ethane for 5 s. The frozen SiN window was transferred into the liquid nitrogen while avoiding exposure to air.

To evaluate if the poisoning intervention shows a biological impact on the viability of *E. coli* cells, optical fluorescence microscopy was performed using the LIVE/DEAD BacLight Bacterial Viability Kit (Invitrogen, L7012; see Fig. S1 of the supporting information). Counting live (green fluorescence) and dead (red fluorescence) cells resulted in yielding a percentage of live *E. coli* cells, demonstrating that ethanol or sodium azide poisoning can deteriorate *E. coli* viability (Table S1 of the supporting information, Fig. S2). Given that poisoning may alter the biodistribution of elements in a single *E. coli* cell, the clustering of type A, B and C samples observed with XRF microscopy images proceeded regardless.

### 2.2. Data acquisition

The XRF elemental maps were acquired at the 9-ID beamline of the APS using the Bionanoprobe. The BNP is an X-ray microscope with nanometre-level probe size and a cryogenic sample environment. The BNP is located at an undulator of the APS synchrotron storage ring. The operating pressure and temperature are below 10^−7^ torr and 135 K. Analysis of trace elements in biological samples at very low temperatures and at high spatial resolution is the key use of the BNP. To maintain cryogenic temperatures, samples are frozen using liquid-nitrogen conductive cooling. The low pressure mitigates frosting and air absorption and also prevents the sample from breaking. The vacuum ensures minimal X-ray absorption in air. Samples are mounted using a precision cryo-robot stage system. The BNP performs raster scans with a 0.25 µm step size and 0.15 µm beam spot size. The scanning speed along the horizontal axis is between 10^−4^ and 10^−2^ mm s^−1^. From the synchrotron storage ring, the generated X-ray beam is monochromated using a double-crystal monochromator and focused using a Fresnel zone plate onto the cryogenic sample. The source X-ray energy for this data was 10 keV. As the sample is raster-scanned, its XRF spectrum is recorded for every pixel using a seven-element Vortex-ME7 energy-dispersive silicon drift detectors (Hitachi High Technologies Science America, USA). The detector is orientated perpendicular with respect to the incident X-ray beam to generate elemental maps of the sample in 2D (Nietzold *et al.*, 2018 ▸).

Dwell time is the amount of time each pixel is exposed to the X-ray beam for the collection of the elemental map. Long dwell times constitute a significant bottleneck for faster experimentation and thus reduction of dwell time is of interest. Five of the coarse scans reported in this paper had a dwell time per pixel of 100 ms. Three coarse scans were conducted at 50 ms, 25 ms and 12.5 ms to understand how much reduction in dwell time is achievable for automated ROI finding.

The elemental maps are formed from the fluorescence counts at different X-ray energies measured from the detector on a raster grid of scan points. In total, eight coarse scans were collected at the BNP in this paper. Five scans consisted of only type A and B cells. Three additional scans of type C cells were collected to analyze the effects of dwell time. The effect of dwell time is discussed later. The elemental maps of these scans are given in the supporting information.

In Table 1 ▸, the sample types, scan size, extracted regions, extracted cells and chemical reagent for sample preparation are given.

## 3. Software

### 3.1. Raw-data format and user inputs

XRF microscopy map images in the .h5 file format containing chemical composition and elemental distribution in a biological sample are loaded after processing from the *MAPS* data-fitting software (Nietzold *et al.*, 2018 ▸). Measured elemental distribution maps are selected by the user depending on the problem. For the current problem, in order to distinguish between type A and B cells, the amount of potassium is crucial since it is expected to be very high in cells that were frozen in a live state with an intact inner membrane. Since K levels are low outside the cell, any physiological disruption of the inner membrane will lead to leakage and lower levels of intracellular K. We anticipate that the cellular content of K in particular will vary depending on the sample preparation steps and treatment which lead to physiological stress for the *E. coli* cells (Matsuyama *et al.*, 2010 ▸; Perrin *et al.*, 2015 ▸). Hence, the choice of K is a crucial factor in distinguishing cell treatment types. Maps of Fe, Zn, Ca and P contrasted well from the background and thus were also selected. Maps of Cu, Ni and Cl were noisy and were difficult to distinguish from the background and thus not selected. For any other scenarios, users may choose any subset of the elemental maps of their interest using the *ROI-Finder* programming interface.

### 3.2. Segmentation and detection of individual cells

**Figure 2:**
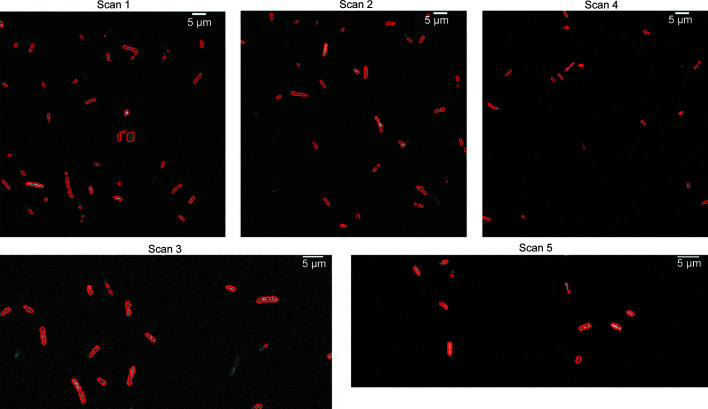
Segmentation masks of the coarse scans containing type A and B cells. Red indicates the boundary between the cell and background regions as detected by the segmentation scheme. Pixels inside the cells are labeled 1 and the background is labeled 0.

The segmentation masks of the five coarse scans containing type A and B cells are illustrated in Fig. 2 ▸. In the mask, all pixels inside the detected cell boundary were labeled 1 and the background was labeled 0. In the segmentation mask we observed the cell boundaries and some small image-processing artifacts during binary conversion via Otsu thresholding.

A connected components filter is applied to the binary image to extract all pixel-wise connected ROIs using the *Scikit-image* package (Van der Walt *et al.*, 2014 ▸). Afterwards, we loop over each ROI performing morphological measurements and fluorescence counts. Some detected ROIs are not *E. coli* cells but image-processing artifacts. To remove them, we apply a selection criteria to accept an extracted ROI as a cell based on the total number of pixels in the region/component. We found that rejecting any region/component containing less than or equal to 8 pixels removes image-processing artifacts. Finally, the features extracted from the ROIs selected are used for the PCA in the next step. For this case, we calculated two morphological features, namely the area and the eccentricity. The elemental features defined from K, P, Ca, Zn and Fe channels are calculated from their maximum amounts calculated over all pixels within an individual ROI.

In order to segment the ROIs (*i.e.* *E. coli* cells) from the elemental maps, the sum of K, P, S and Ca channels is used to construct a single coarse image. A median filter with a 3 × 3 kernel is applied and then the image is thresholded via Otsu thresholding to create a binary segmentation mask (Otsu, 1979 ▸). We used 1.25× the Otsu threshold value for segmentation. In order to quickly preview and adjust parameters for generating the segmentation masks, *Jupyter Notebooks* are provided.

### 3.3. Principal component analysis

**Figure 3:**
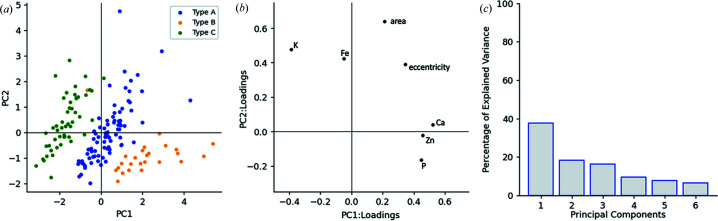
(*a*) PCs and (*b*) associated loading scores on the extracted cells from the eight coarse scans shown in Figs. 2 ▸ and 5 ▸. (*c*) Scree plot. Type A, B and C cells are clustered in the PC space due to their morphological and maximum elemental mapping variation. Each extracted feature and their direction of variation are depicted by the loading scores plot. The scree plot illustrates how much variation in the data is explained by each PC.

In Fig. 3 ▸, all single-cell data extracted from the eight coarse scans are shown in a single scatter plot along with the associated loadings. The PCA method is described in the next section. We plotted two PCs for ease of visualization, but the scree plot indicates that at least six PCs are required to fully explain the variance in the data. For complex tasks, attempting to cluster or classify more than three types of cell, more descriptive features may be needed and thus users may need to plan and carefully gather data consisting of higher-dimensional space consisting of more than seven features and/or consider more than two PCs.

It is apparent from the scatter plot of the first two PCs that there are distinct clusters for *E. coli* cells that were treated differently, originating from three different sets of sample preparation techniques. Since more PCs are required to fully capture the variance in the entire dataset, and we are already seeing clusters appearing in 2D, we expect to form stronger clusters in a higher-dimensional space if we include more PCs in our analysis.

In this work, the PCs were constructed from seven features. The equations for the first two PCs are given below where *a* represents the area; *e* represents the eccentricity of the *E. coli* cells; and K, P, Ca, Zn and Fe represent the maximum fluorescence count for these elements present in the *E. coli* cells. Extracting cell-averaged fluorescence counts from elemental maps did not demonstrate improved clustering compared with the extraction of maximum fluorescence counts from the elemental maps for elemental feature construction. This may be problem specific, and for other clustering applications for scientific discovery, cell-averaged fluorescence counts from elemental maps may yield improved clustering. *ROI-Finder* allows the user to extract fluorescence counts in both ways and provides functionalities so the user may define their own type of extraction of fluorescence counts from the elemental maps. The fluorescence counts can also be converted to a concentration value. While this may affect the magnitude of calculated PCs, their direction should remain unchanged and thus the relative position of the cluster points in the PC space should be preserved since each PC consists of a scaling component (eigenvalues) and a direction component (eigenvectors),

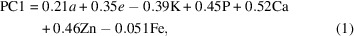




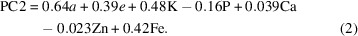

The loading scores of each of these features indicate their direction of variation. This analysis distinguishes between cells based on the treatment groups. We find that type C cells are distinguished by higher amounts of K compared with the other two types. Similarly, type B cells contain more Ca, Zn and P relative to type A and C cells. The variation of morphology between the cells can mostly be attributed to the variation of the elemental features. We note that very large *E. coli* cells are predominantly outliers and are located far away from cluster centers in the first quadrant. These are likely to be dividing cells (two cells very close together) or scanning artifacts (parts of the scan were corrupted). From the morphological features, it is apparent that the cells of each type have good morphological variation and a wide range of sizes.

PCA produces a set of successive orthogonal variables from correlated high-dimensional data to explain the maximum variance in the data (Abdi & Williams, 2010 ▸). In this instance, the multi-variate data consist of seven features extracted from the regions/components in the previous step, namely area (*a*); eccentricity (*e*); and maximum fluorescence count (calculated for all pixels within an ROI) of K, P, Ca, Zn and Fe elements. The input to the PCA method is thus a high-dimensional data table consisting of these features. PCA was performed using the *Scikit-learn* Python package. The data input for PCA is represented as a matrix **X** of *n* × *f* dimensions where *n* is the number of rows/cells/ROIs and *f* is the number of columns/features. The data were standardized by subtracting the mean and normalized by the standard deviation. The covariance matrix (**C**) is given by 

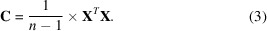

Since **C** is symmetric it can be written as

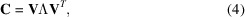

where **V** is the eigenvector matrix and Λ is a diagonal matrix with progressively decreasing eigenvalues. The eigenvectors are the principal axes/directions of the data. Projections of the data on the principal directions are PCs and are given by the columns of **XV** or **US**, where **U** is a unitary matrix and **S** is the singular matrix when PCA is performed with singular value decomposition (SVD) and **X** = **US** **V** ^T^. The ‘loadings’ of the PCs are given by [1/(*n* − 1)] × **VS**.

### 3.4. Clustering and similarity-based recommendation

We further perform cluster analysis on the loadings derived from the PCA step. Here, we use PCA as a dimensionality reduction step to select the first two PCs for cluster analysis.

*Manual selection of clusters*. As a first step, one may want to manually tag the cells represented as scatter points in the PC plot space. If annotations are available, the scatter points will clearly demonstrate the differences between the ROIs/cells. In the absence of annotations, users can apply various filters on the data based on heuristics to better understand the trends present in the ROIs/cells. Experiment-to-experiment variation can also be better understood by progressively adding ROIs/cells from each experiment as they are measured in the PC space plot. The user can view the clustering assignments to gain further understanding. When the user chooses an ROI/cell using a lasso-selector tool, the nearest ROI/cell to that cluster is recommended for fine scanning.

*Similarity-based sorting with fuzzy k-means*. Selection of clusters does not need to be carried out manually. An automated clustering algorithm could assign the ROIs/cells to different integer class labels without manual classification. Given *n* cells with PCs as features *x*_*i*_, we want to group *n* cells into *k* number of clusters **C** where no cell is placed in more than one cluster. The *k*-means clustering algorithm minimizes the within-cluster sum of squares criterion for each cell *x*_*i*_ (Barbakh *et al.*, 2009 ▸). Once the class labels are available from *k*-means for two clusters to distinguish between type A and B cells, *ROI-Finder* can recommend cells of each type from the assigned cluster of cells detected in a coarse scan for a follow-up fine scan,

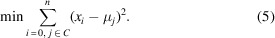

In the *k*-means clusters, each ROI or cell is strictly assigned to one cluster. Ideally, all type A cells should closely cluster around a cluster center. Similarly, all type B cells should also form a cluster around its own cluster center. There could be borderline cases when an ROI or cell is placed at an approximately equal distance from multiple cluster centers. In this instance it is difficult to identify the ideal cluster center for that particular ROI or cell. However, the hard clustering *k*-means algorithm would assign these ROIs to a single cluster since there will always be a difference in the measured distances from the clusters, regardless of how small they are. Even for human experts, assigning these borderline cases to the correct cluster centers or identifying the treatment type would be difficult. Such cases are also likely to arise due to experimental anomalies, poor contrast of ROIs from the background and low signal-to noise ratio (SNR). Thus a metric is required to assign ROIs/cells to more than one cluster or provide an indication of how likely they are to belong in one cluster compared with the others. Fuzzy *k*-means clustering accomplishes this by providing this weight-based metric to assess the clustering and rank/sort the cells for each individual cluster (Li *et al.*, 2017 ▸). In fuzzy *k*-means clustering, the following function is minimized with the associated weights *w*_*ij*_ ∈ [0, 1],

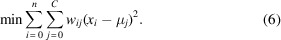

The weights are given by

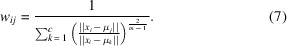

Here, *m* is a softness/fuzziness parameter and is set to 2; *m* = 1 indicates hard clustering. The confidence metric refers to the largest weight belonging to a cluster.

The learning of the weights from the training data *x*_*i*_ is a key step in the implementation of machine learning for guiding XRF fine scans.

## 4. Use cases

### 4.1. Clustering type A and B cells

**Figure 4:**
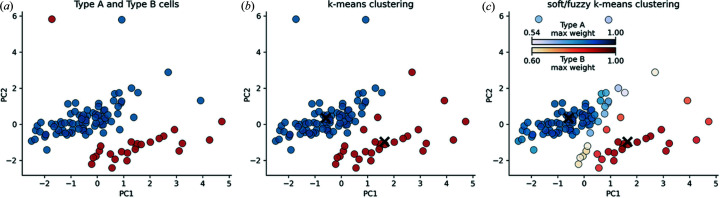
Comparing (*a*) ideal, (*b*) regular and (*c*) soft or fuzzy *k*-means clustering. Blue points are type A cells and red points are type B cells. Each scatter point represents a cell. In plot (*c*), the scatter point color intensity is scaled with the confidence metric for fuzzy clustering. The detected cells with low confidence metrics are barely visible. These cells are probably experimental anomalies. The cluster center is depicted by the ‘X’ marker.

We clustered the 106 cells extracted from coarse scans 1–5 in Fig. 4 ▸ using their PCs. Histograms showing the distribution of values for each feature are provided in the supporting information. *ROI-Finder* uses two PCs for the regular and fuzzy clustering. In Fig. 4 ▸, the ground-truth labels are shown on the left, the regular *k*-means clusters are shown in the middle, and the soft, the result of fuzzy *k*-means clustering, is shown on the right. When the labels associated with each cell are available, we have an annotated dataset, and this can be used to perform conventional supervised classification and any supervised machine-learning model will suffice. For the purpose of testing *ROI-Finder*, scans 1–5 were conducted carefully with samples containing only type A cells for scans 1–3 and only type B cells with scans 4–5 and thus the annotations are available to us.

From the regular *k*-means clustering in Fig. 4 ▸, we observe that the algorithm has correctly assigned two clusters and found reasonable cluster centers, although two of the outlying relatively large cells, located between the space of PC1 = 2 to PC1 = 4 and PC2 = 0.5 to PC2 = 2.5, are incorrectly clustered. The outlier between the space PC1 = −1 to PC1 = −2 and PC2 = 5 to PC2 = 6 is also incorrectly clustered by regular *k*-means. The fuzzy *k*-means plot was prepared by tagging all the cells evaluated to a confidence metric below 0.99. The confidence metric is the maximum weight calculated for each cluster. If the *k*-means algorithm associated a higher weight to a cell to cluster it, then this indicates that the algorithm is confident in the cluster assignment. Thus, we can remove all the cells that have very low confidence metrics and leave only the cells that the algorithm is highly confident about clustering. We can further observe that most of the outliers have low weights and are thus poorly clustered; hence, as we move further from the cluster centers, the clustering is likely to be less correct. The advantage of filtering the cells with low confidence values is that it gives the user the opportunity to quickly examine borderline cells/ROIs to better interpret their experimental data. Scanning artifacts or any other artifacts due to experimental design are also likely to have lower confidence values and thus can be easily identified during beam time and users may choose to correct the experimental conditions to address the anomalies.

#### 4.1.1. Codeblock

An example Python code is given in the supporting information to demonstrate how *ROI-Finder* can be integrated with the BNP/EPICS control codes.

### 4.2. Recommending similar cells

*ROI-Finder* can recommend cells similar to those selected by the user via a lasso-selector tool from the regular *k*-means clusters shown in Fig. 4 ▸. To recommend a similar cell, the nearest-neighbor cell measured by the Euclidean distance in the 2D PC space is returned.

### 4.3. Effect of dwell time

**Figure 5:**
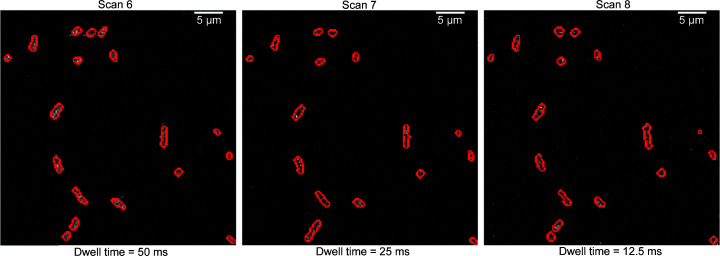
Segmentation masks of the coarse scans containing type C cells. Red indicates the boundary between the cell and background regions as detected by the segmentation scheme. Pixels inside the cells are labeled 1 and the background is labeled 0.

In Fig. 5 ▸, segmentation masks of the coarse scans containing type C cells are shown. The same area of the sample was used for all these three scans. In XRF experiments, reducing the dwell time for faster experimentation introduces other challenges. While longer dwell times in scans 6 (50 ms) and 7 (25 ms) provide good SNRs, we found that, by using K, P, S and Ca channels together as intensity maps to detect individual cells, we can use a shorter dwell time of 12.5 ms to identify cells from noisy scans. The SNRs of scans 6, 7 and 8 with respect to the binary mask of scan 6 are 1.64, 0.97 and 0.80. While the SNR values decrease significantly as dwell times are decreased, it is evident from the binary masks that most of the cells can still be identified. With dwell times of 25 ms and 12.5 ms compared with 50 ms, we lose one cell in the top portion of the binary masks.

### 4.4. Effect of proportion of type A and B cells to the total data on clustering

In Table 2 ▸, calculated dice scores for subsets of the data are given. The dice score corresponds to the accuracy with which the ROIs are classified into their respective treatment groups when compared with the ground-truth labels. Each row of the table represents 1000 random samplings of a specific proportion of cells of each type for clustering. The results are then clustered and the corresponding dice score is calculated for all 1000 samplings. The average dice score improves when the number of cells clustered from each type increased. Increasing the training images allows the clustering algorithm to learn diverse patterns present in the data through the variability present in the larger data and thus the accuracy of the clustering increases.

The dice scores (DS) for each of these clusters are calculated as follows,

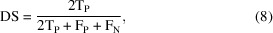

where, *T*_P_, *F*_P_ and *F*_N_ refer to true positives, false positives and false negatives, respectively.

## 5. Conclusions and future work

The generalizability of the *ROI-Finder* to more than two classes depends on the efficiency of the chosen clustering algorithm. With increasing number of classes or types of ROIs/cells/experiments, more data would be needed to efficiently minimize the loss function of the clustering algorithm. The use of the mean-shift clustering algorithm is also recommended in such cases to obtain a preliminary guess to unveil the expected number of clusters in the data before using *k*-means or any other clustering algorithm. The current *ROI-Finder* is developed specifically for use in the BNP (currently located at the APS beamline 9-ID), but the ideas presented in this paper can be extrapolated to use this as a template and adjust it according to the experimental needs of other users. To make the *ROI-Finder* more generalizable so it can be used for other types of cells such as HeLa cells or mouse cells, the segmentation of cells from the microscopy images is very important. If the cells/ROIs can be segmented and extracted, relevant features for PCA can be defined and subsequently *ROI-Finder* can identify the relevant ROIs.

With more variation in the data or if the size of the data is large, more PCs may be needed to represent the variation in the data. If the number of features or the number of cells is very high, use of trained unsupervised deep neural networks may be required to extract relevant features and used for clustering.

If the ROIs/cells contain significant variation, a smaller number of cells can be clustered via PCA and the *k*-means algorithm. As shown in Table 2 ▸, we observe that, even with a smaller number of cells, *k*-means performs useable clustering of two types of cells. So, *ROI-Finder* is useable with smaller amounts of data and without manual annotations.

We describe machine-learning guided *ROI-Finder* and demonstrate its utility in single-cell analysis of XRF microscopy data. The *ROI-Finder* automatically segments images based on relevant elemental maps. Based on limited domain knowledge, morphological and elemental content features are extracted from the elemental maps. Fuzzy *k*-means clustering is then used on calculated PCs to cluster the data and reveal how the elemental content of cells change as a function of cell treatment. The nearest cells in the clustering space to a target region are recommended for a finer scan. The software provides real-time feedback of statistical information that guide subsequent XRF scans. These efforts are expected to address the challenges of the upcoming upgrade to the APS-U and meet subsequent user needs.

Data will be provided upon request (Github repository: https://github.com/aisteer/ROI-Finder).

## 7. Related literature

The following references, not cited in the main body of the paper, have been cited in the supporting information: Boulos *et al.* (1999 ▸); Robertson *et al.* (2019 ▸); Stiefel *et al.* (2015 ▸).

## Supplementary Material

Optical fluorescence microscopy sample preparation procedure and histograms of the extracted cell features. DOI: 10.1107/S1600577522008876/rv5165sup1.pdf

## Figures and Tables

**Table 1:** Characteristics of the coarse scans collected at the 9-ID bionanoprobe of the APS All coarse scans were collected under cryogenic conditions and have a resolution of 0.25 µm.

Scan index	Sample type	Scan size	Dwell time (ms)	Extracted regions†	Extracted cells	Chemical reagent
1	A	321 × 321	100	41	36	Untreated
2	A	321 × 321	100	36	31	Untreated
3	A	149 × 321	100	17	14	Untreated
4	B	321 × 321	100	19	17	70% ethanol
5	B	118 × 321	100	11	8	70% ethanol
6	C	163 × 163	50	18	18	1 m*M* NaN_3_ + 0.1 m*M* CuSO_4_
7	C	163 × 163	25	16	16	1 m*M* NaN_3_ + 0.1 m*M* CuSO_4_
8	C	163 × 163	12.5	17	16	1 m*M* NaN_3_ + 0.1 m*M* CuSO_4_

† These extracted regions are based on the scheme described later.

**Table 2:** Average dice scores from 1000 randomly sampled cases For each case a certain percentage of cells was selected from each type of cells and clustered.

Percentage	Dice score	Type A cells	Type B cells	Total cells
35	0.64	29	9	38
45	0.66	37	12	49
55	0.69	45	14	59
65	0.75	53	17	70
75	0.79	61	19	80
85	0.86	69	22	91
