# Supplementary material for: ROI-Finder: machine learning to guide region-of-interest scanning for X-ray fluorescence microscopy
Source: J Synchrotron Radiat. 2022 Oct 25;29(Pt 6):1495–503. doi: 10.1107/S1600577522008876 (PMC9641565; doi:10.1107/S1600577522008876)
Supplement: Supplementary file 1 [file s-29-01495-sup1.pdf]

JOURNAL OF SYNCHROTRON RADIATION

Volume 29 (2022)

Supporting information for article:

ROI-Finder: machine learning to guide region-of-interest scanning for X-ray fluorescence microscopy

M. A. Z. Chowdhury, K. Ok, Y. Luo, Z. Liu, S. Chen, T. V. O'Halloran, R. Kettimuthu and A. Tekawade

Supplementary material to XRF-ROI-Finder: Machine learning to guide region-of-interest scanning for X-ray fluorescence microscopy

M.A.Z. Chowdhury,[a] K. Ok,[b] Y. Luo,[c,d] Z. Liu,[a] S. Chen,[c,d]
T.V. O'Halloran,[b,e] R. Kettimuthu[a] and A. Tekawade [a*]

[a]*Data Science and Learning Division, Argonne National Laboratory, Lemont, IL 60439, USA,* [b]*Department of Microbiology and Molecular Genetics, Michigan State University, East Lansing, MI 48824, USA,* [c]*X-ray Science Division, Argonne National Laboratory, Lemont, IL 60439, USA,* [d]*Advanced Photon Source, Argonne National Laboratory, Lemont, IL 60439, USA, and* [e]*Department of Chemistry, Michigan State University, East Lansing, MI 48824, USA.*

E-mail: atekawade@anl.gov

microscopy; **X-ray fluorescence**; **Escherichia coli**; **fuzzy clustering**; **region-of-interest**

1. *E. coli* viability test via optical fluorescence microscopy.

To verify that *E. coli* cells were poisoned by 70% ethanol (Type B) or 1 mM sodium azide + 0.1 mM copper sulfate (Type C) treatment, *E. coli* viability test was performed using optical fluorescence microscopy. The LIVE/DEAD BacLight^{TM} Bacterial Viability Kit (Invitrogen, L7012) enables a detection of live and dead bacterial cells using fluorescent dyes, SYTO9 (excitation/emission: 480/500 nm) and propidium iodide (PI, excitation/emission: 490/635nm) (Boulos et al., 1999). The SYTO9 green-fluorescent nucleic acid stain labels all *E. coli* cells with intact and damaged membranes, while PI red-fluorescent nucleic acid stain penetrates only *E. coli* cells

with damaged membranes and reduces the SYTO9 stain fluorescence when both dyes are present (Stiefel et al., 2015; Robertson et al., 2019). *E. coli* sample preparation protocol for the optical fluorescence microscopy is as follows. A single colony of *E. coli* K-12 wild type strain MG1655 was inoculated into 5 mL of sterilized LB broth (Miller's modification, Sigma Aldrich, L3522) and incubated overnight (16 hours) at 37°C with 250 rpm shaking. The overnight culture was diluted to 1:100 in three tubes containing 25 mL of fresh LB broth media and grown for 1.5 hours to reach early-log phase (O.D.$_{600}$ = 0.3-0.4). The *E. coli* cells were then exposed to three different treatments and incubated for additional 20 minutes. Type A: healthy untreated (control), Type B: 70% ethanol (200 proof, Decon Labs Inc., 2701) treated, Type C: 1 mM sodium azide (Sigma Aldrich, S2002) + 0.1 mM copper sulfate (Sigma Aldrich, C8027) treated. For Type B sample, the LB medium was replaced to 70% ethanol solution and incubated. All samples were washed three times with 240 mM sucrose (Sigma Aldrich, 84097) solution by centrifugation at 10,000 x g for 10 min at room temperature. An O.D.$_{600}$ of washed *E. coli* suspension was adjusted to 0.9-1.0. For optical fluorescence microscopy, a mixture solution of both fluorescent dyes (1.7 mM SYTO9 + 10 mM PI dye) was added to the cell suspension at a volume ratio of 1 to 350 and incubated for 15 minutes in the dark at room temperature. Nikon Eclipse Ti-U fluorescence microscope equipped with Nikon S Plan Fluor ELWD 40XC lens and filter set (light source: CoolLED pE-300white) was utilized to obtain the green and red fluorescence images of stained *E. coli* cells. Green (live) and red (dead) fluorescence labeled *E. coli* cell images on the same focal plane were merged (Figure S1). To yield a percentage of live bacteria (live cells/total cells), 10 images per each type of sample were collected, and green and red cell number of each image was counted using the particle analysis function of the ImageJ software (Table S1, Figure S2). Experiments were performed in triplicate for n=30.

Table 1. *Poisoned E. coli cell viability test results (n=30)*

Sample	Treatment	Percent Live Bacteria (%, Mean ± SD)
Type A	Healthy untreated (control)	90 ± 6.6
Type B	70% Ethanol	1.1 ± 1.7
Type C	1 mM NaN_3 + 0.1 mM $CuSO_4$	67 ± 13

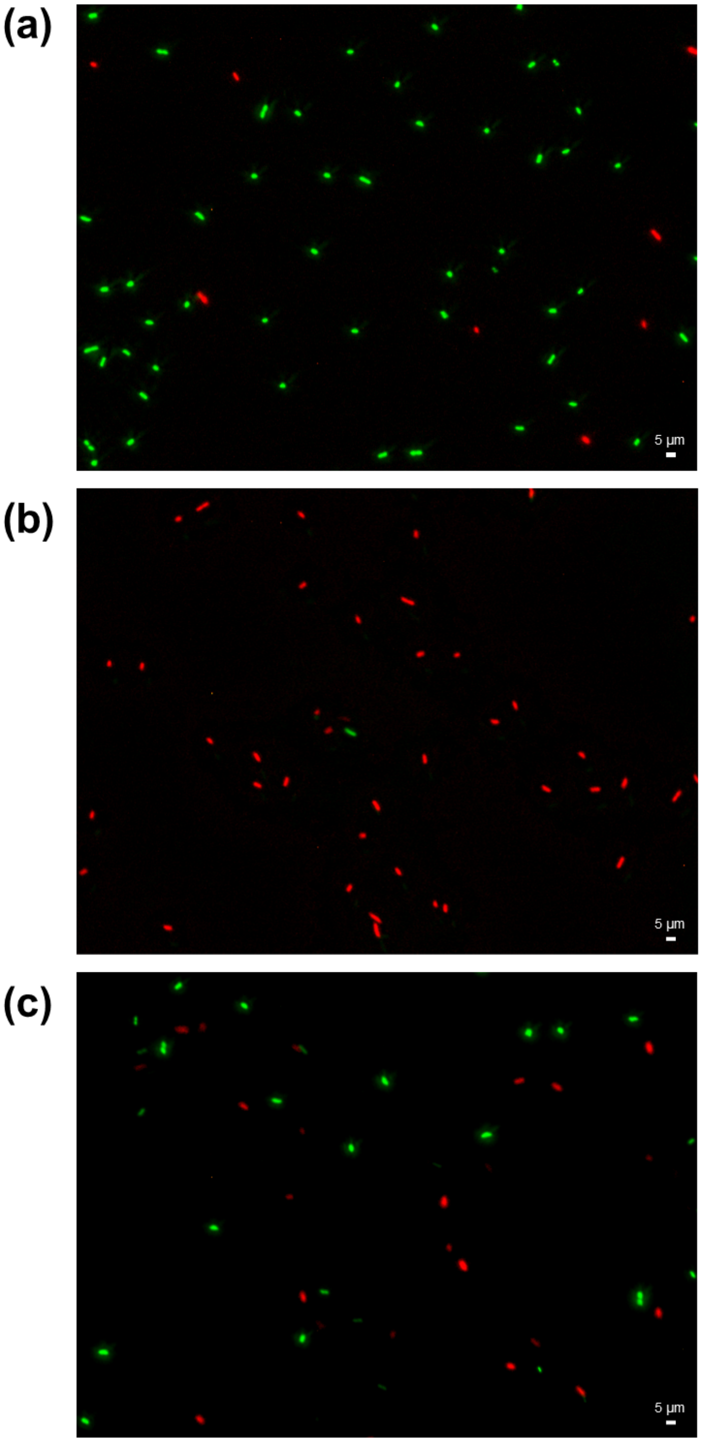

Fig. 1. Visualized fluorescence images of live (green) and dead (red) *E. coli* cells using LIVE/DEAD BacLight^{TM} Bacterial Viability Kit (Invitrogen, L7012). (a) Type A sample: Healthy untreated (control), (b) Type B sample: 70% Ethanol treated, and (c) Type C: 1 mM sodium azide (NaN_3) and 0.1 mM copper sulfate ($CuSO_4$) treated.

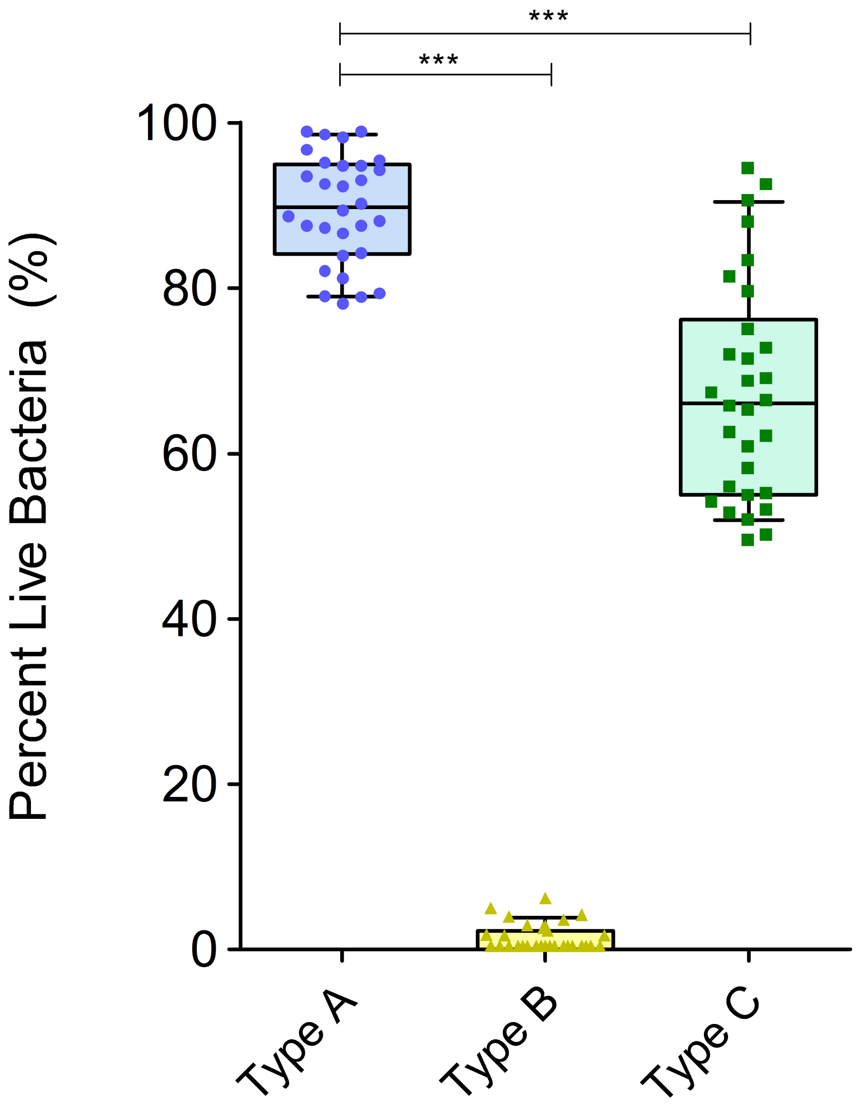

Fig. 2. Poisoned *E. coli* cell viability test results using LIVE/DEAD BacLight^{TM} Bacterial Viability Kit. Live and dead cells per image were counted to yield a percentage of live bacteria (live cells/total cell number). Type A: Healthy untreated (control), Type B: 70% Ethanol treated, Type C: 1 mM sodium azide (NaN_3) and 0.1 mM copper sulfate ($CuSO_4$) treated. [Box-and-whisker plot: 10-90 percentile (median), ***: p <0.0001, n=30]

2. X-ray fluorescence microscopy elemental maps for all the scans.

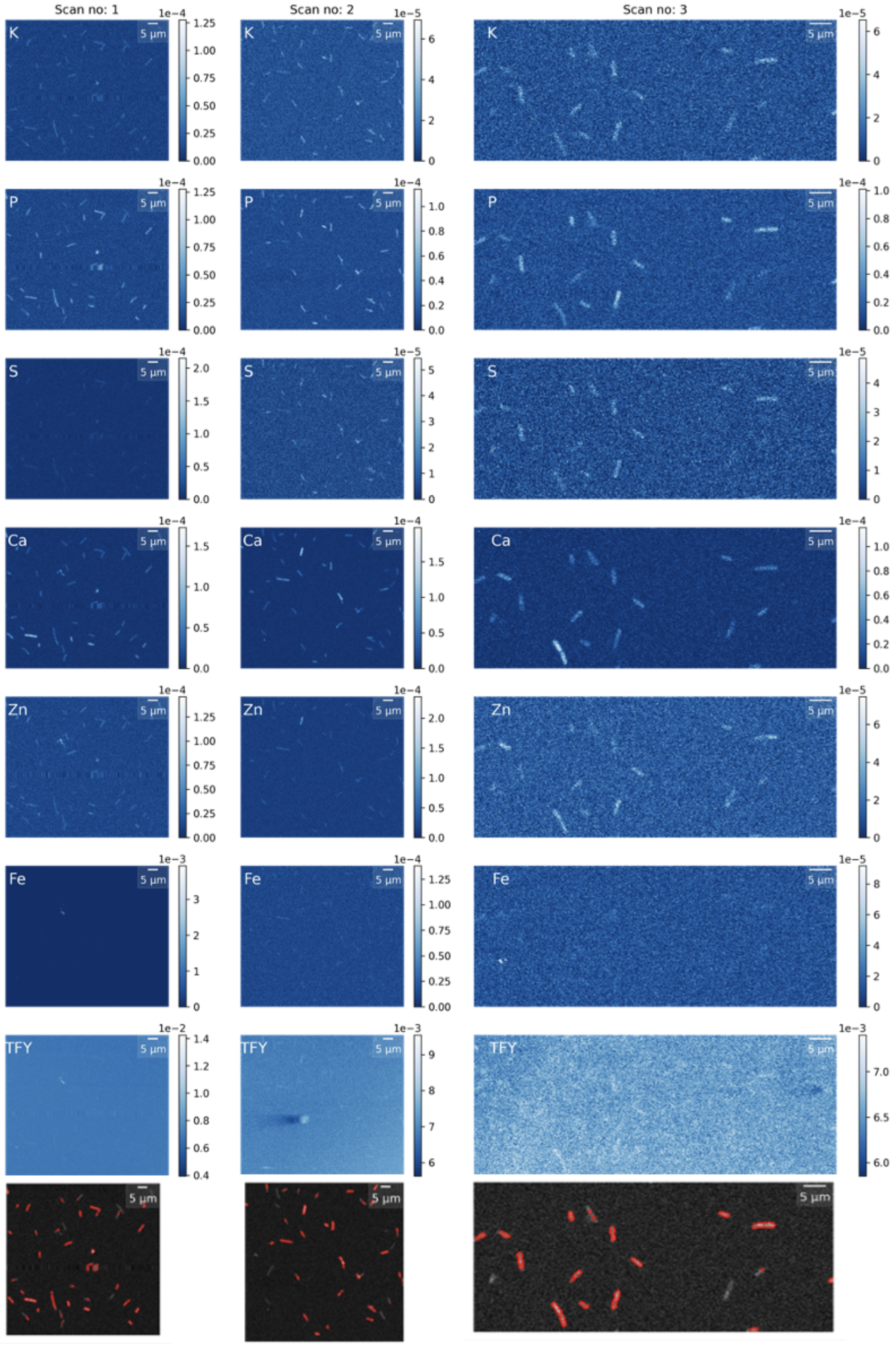

Fig. 3. Elemental maps and segmentation for Type A *E. coli* samples

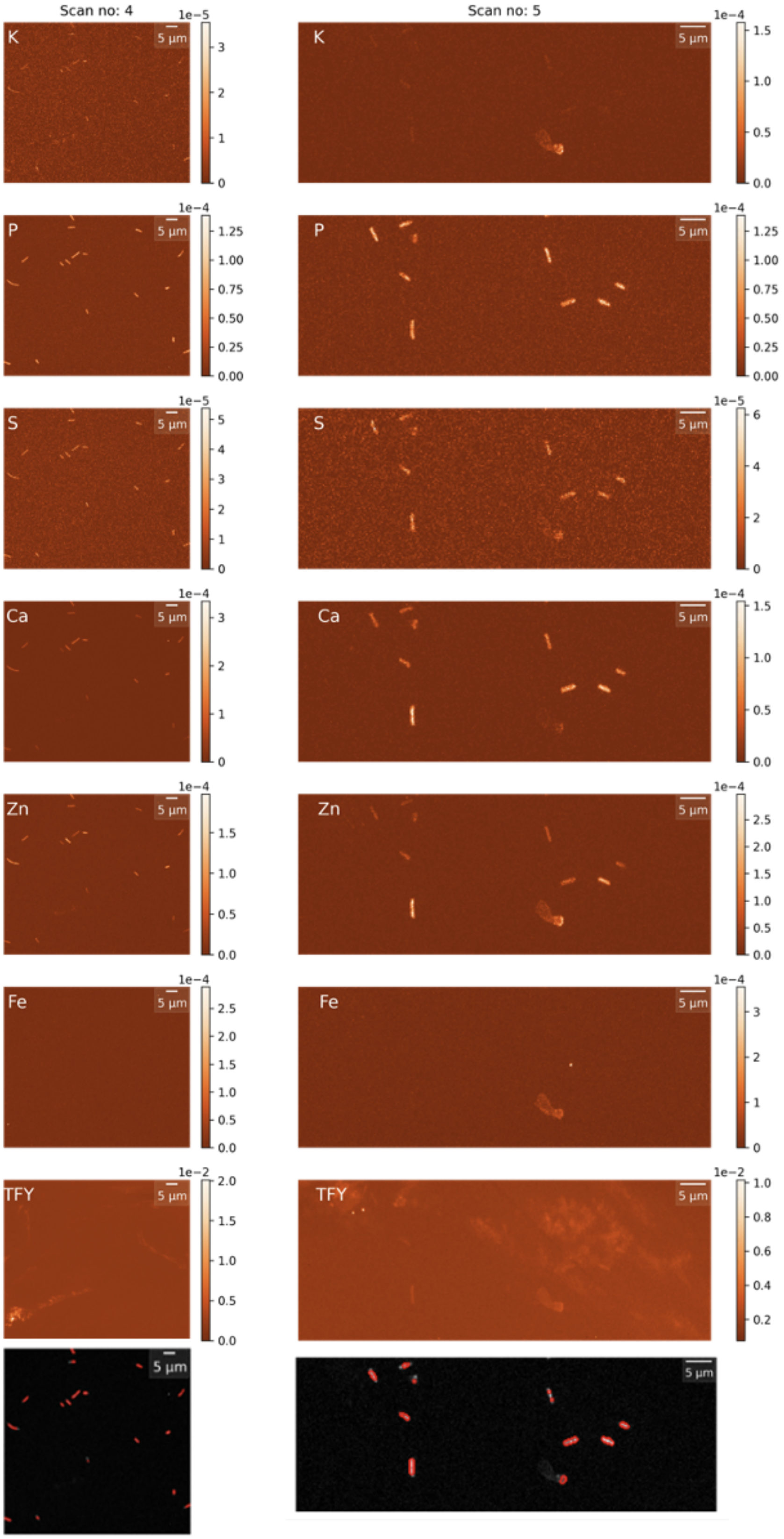

Fig. 4. Elemental maps and segmentation for Type B *E. coli* samples

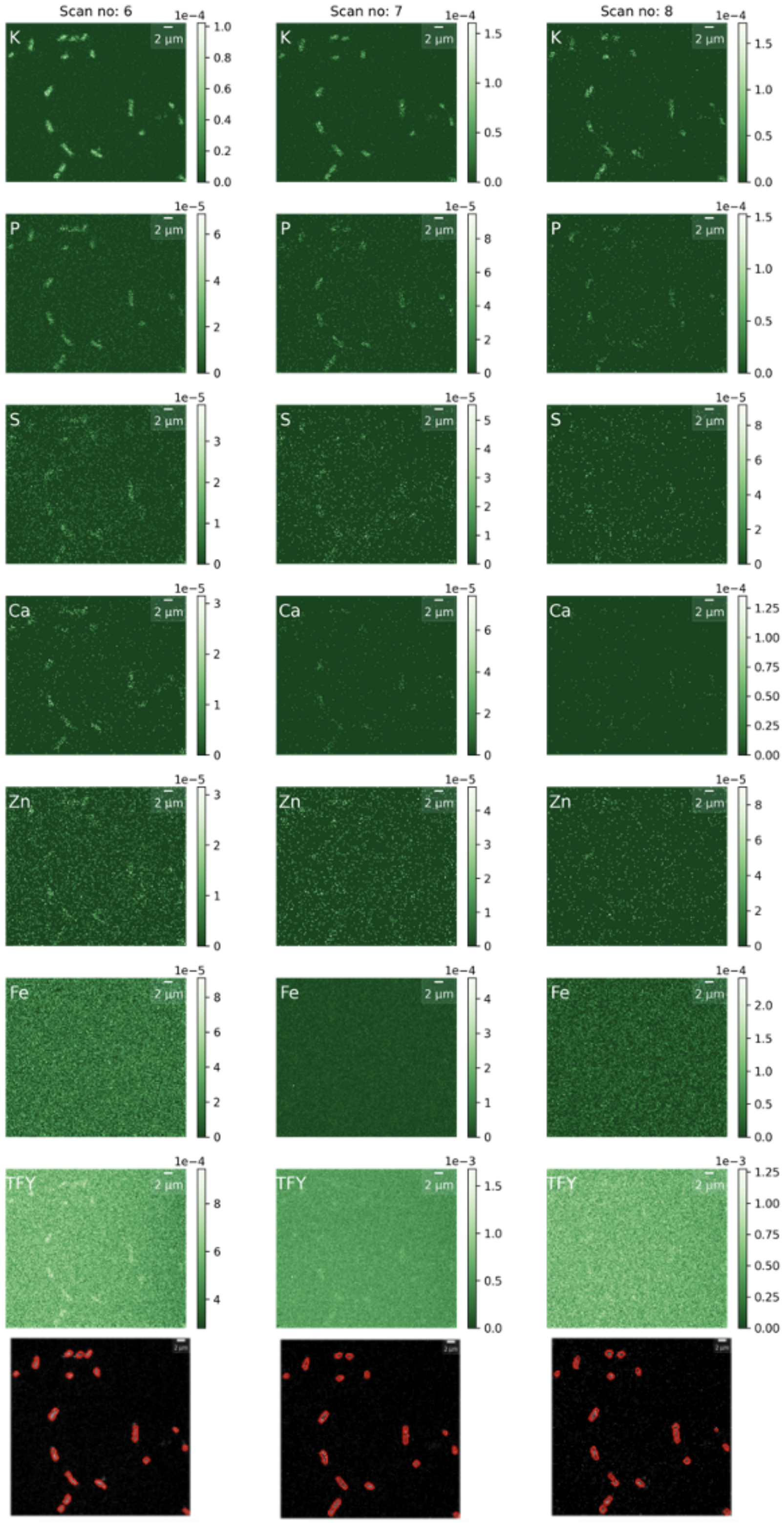

Fig. 5. Elemental maps and segmentation for Type C *E. coli* samples

3. Histograms of the features of the extracted *E. coli* cells

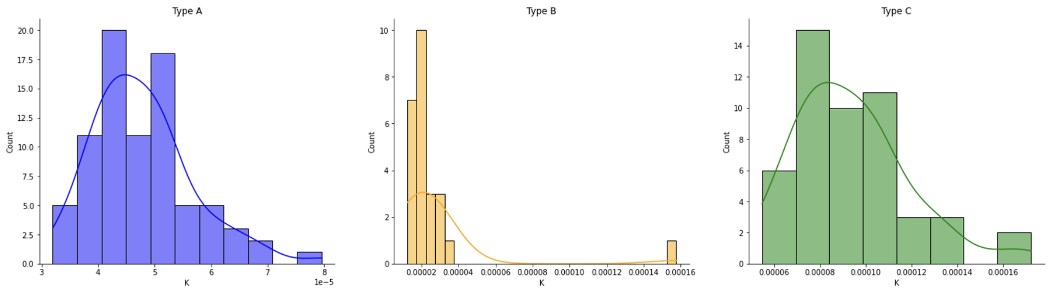

Fig. 6. Histograms of the maximum amount of potassium feature.

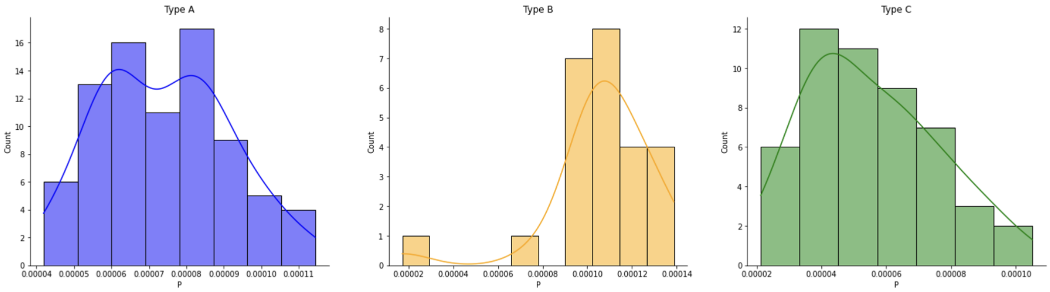

Fig. 7. Histograms of the maximum amount of phosphorus feature.

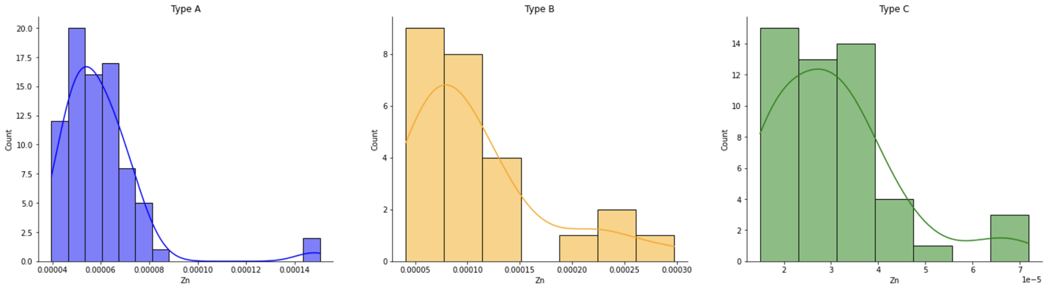

Fig. 8. Histograms of the maximum amount of zinc feature.

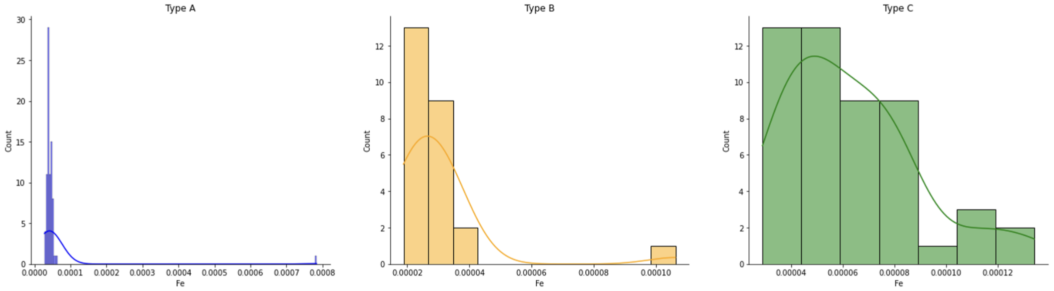

Fig. 9. Histograms of the maximum amount of iron feature.

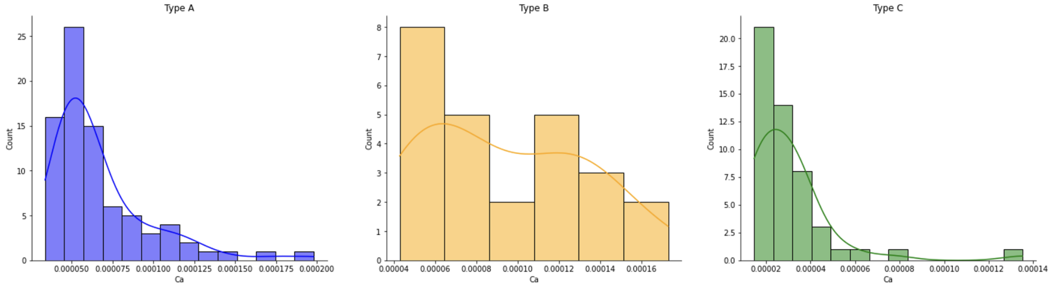

Fig. 10. Histograms of the maximum amount of calcium feature.

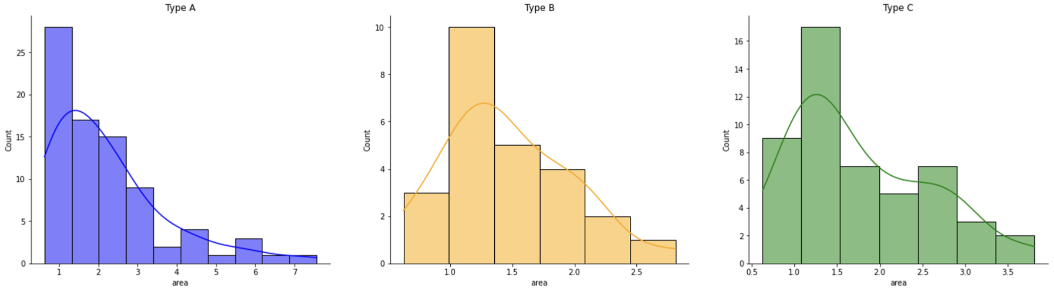

Fig. 11. Histograms of the maximum amount of area feature.

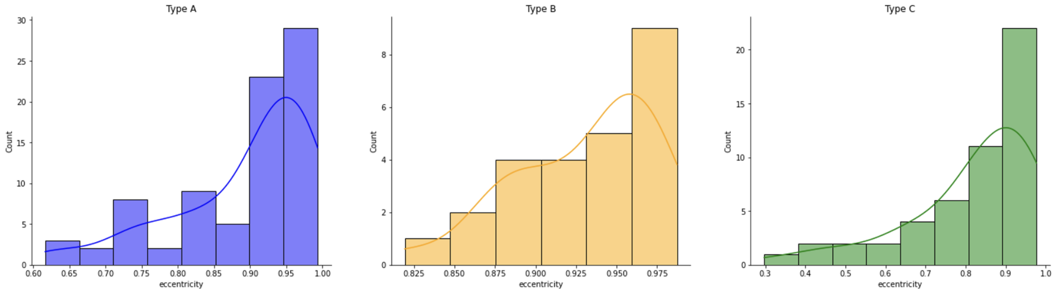

Fig. 12. Histograms of the maximum amount of eccentricity feature.

References

Boulos, L., Prevost, M., Barbeau, B., Coallier, J. & Desjardins, R. (1999). Journal of microbiological Methods, **37**(1), 77–86.

Robertson, J., McGoverin, C., Vanholsbeeck, F. & Swift, S. (2019). Frontiers in microbiology, **10**, 801.

Stiefel, P., Schmidt-Emrich, S., Maniura-Weber, K. & Ren, Q. (2015). BMC microbiology, **15**(1), 1–9.
